# Identification and Characterization of Multiple TRIM Proteins That Inhibit Hepatitis B Virus Transcription

**DOI:** 10.1371/journal.pone.0070001

**Published:** 2013-08-01

**Authors:** Shijian Zhang, Ju-Tao Guo, Jim Z. Wu, Guang Yang

**Affiliations:** 1 Roche R&D Center China LTD, Pudong, Shanghai, China; 2 Shanghai Medical College of Fudan University, Shanghai, China; 3 Institute for Biotechnology and Virology Research, Department of Microbiology and Immunology, Drexel University College of Medicine, Doylestown, Pennsylvania, United States of America; Yonsei University, Republic of Korea

## Abstract

Tripartite motif (TRIM) proteins constitute a family of over 100 members that share conserved tripartite motifs and exhibit diverse biological functions. Several TRIM proteins have been shown to restrict viral infections and regulate host cellular innate immune responses. In order to identify TRIM proteins that modulate the infection of hepatitis B virus (HBV), we tested 38 human TRIMs for their effects on HBV gene expression, capsid assembly and DNA synthesis in human hepatoma cells (HepG2). The study revealed that ectopic expression of 8 TRIM proteins in HepG2 cells potently reduced the amounts of secreted HBV surface and e antigens as well as intracellular capsid and capsid DNA. Mechanistic analyses further demonstrated that the 8 TRIMs not only reduced the expression of HBV mRNAs, but also inhibited HBV enhancer I and enhancer II activities. Studies focused on TRIM41 revealed that a HBV DNA segment spanning nucleotide 1638 to nucleotide 1763 was essential for TRIM41-mediated inhibition of HBV enhancer II activity and the inhibitory effect depended on the E3 ubiquitin ligase activity of TRIM41 as well as the integrity of TRIM41 C-terminal domain. Moreover, knockdown of endogenous TRIM41 in a HepG2-derived stable cell line significantly increased the level of HBV preC/C RNA, leading to an increase in viral core protein, capsid and capsid DNA. Our studies have thus identified eight TRIM proteins that are able to inhibit HBV transcription and provided strong evidences suggesting the endogenous role of TRIM41 in regulating HBV transcription in human hepatoma cells.

## Introduction

Tripartite motif (TRIM) proteins are RING-type E3 ligases. These proteins share a conserved N-terminal structure consisting of a RING finger domain, one or two B boxes, a putative coiled-coil domain, and a variable C terminus. Based on the structure of C terminus, the TRIM family members can be divided into 11 groups [1]. TRIMs are ubiquitously expressed in many tissues and organs, and involved in a variety of cellular processes, including innate and adaptive immune responses [2]–[4]. Several TRIM proteins are interferon (IFN) inducible and can restrict viral infections [5]. TRIM5α, being one of the most highly implicated TRIM family proteins in cell-intrinsic antiviral immunity, restricts the infection of various retroviruses, such as human immunodeficiency virus 1 (HIV-1) and murine leukemia virus (MLV) [6]–[8]. In addition, TRIM28 is critical for the silencing of endogenous retroviruses in embryonic stem cells [9]. TRIM79α inhibits Tick-borne encephalitis virus replication by degrading the viral RNA polymerase [10]. TRIM22 inhibits the transcriptional activities of hepatitis B virus (HBV) core promoter and HIV-1 LTR [11], [12]. TRIM56 restricts the infection of bovine viral diarrhea virus *via* interaction with the viral N-terminal protease [13]. Moreover, a screen of 55 human and mouse TRIM proteins identified more than 20 TRIMs that inhibited the early or late events of HIV-1 and/or MLV replication cycle [14].

Besides serving as effectors of innate antiviral immune response, multiple TRIMs also regulate the pattern recognition receptor (PRR) signal transduction pathways and thus indirectly modulate viral infection and viral pathogenesis [2], [4]. For instance, TRIM21 negatively regulates the activation of NFκB and IRF3/7 induced by multiple toll-like receptors (TLRs) and RIG-I-like receptors (RLRs) [15]. Lys 63-linked ubiquitination of RIG-I by TRIM25 is essential for optimal induction of RIG-I-mediated IFN response [16]. TRIM30α negatively regulates TLR-mediated NFκB activation by targeting TAB2 and TAB3 for lysosomal degradation [17]. TRIM56 facilitates TLR3 ligand and cytoplasmic double-stranded DNA stimulated IFN induction by interaction with TRIF and ubiquitination of STING, respectively [18], [19]. Recently, a systematic screening of all 75 known human TRIMs revealed that approximately one half of the 75 TRIM-family members enhanced the innate immune response and that they did this at multiple levels in signaling pathways [20]. Moreover, Uchil and colleagues identified 16 TRIM proteins that induced NFκB and/or AP-1. Particularly, TRIM62 was shown to be essential for TRIF-mediated late NFκB, AP-1, and interferon production following lipopolysaccharide stimulation. Interestingly, most TRIM proteins previously identified to inhibit MLV demonstrated an ability to induce NFκB/AP-1 [21].

Hepatitis B virus (HBV) infection is a major public health problem. Currently, it has been estimated that 350 million people worldwide are chronically infected with HBV. Approximately one-third of these individuals will die from serious liver diseases, such as cirrhosis, hepatocellular carcinoma and liver failure, if left untreated [22]. Because HBV infection of hepatocytes induces negligible cytopathic effects, it is generally believed that the outcome of HBV infection and severity of its associated liver diseases are determined by the nature and strength of host innate and adaptive immune responses against the virus [23], [24]. Furthermore, alpha interferon (IFN-α) is approved by administrative authorities for treatment of chronic hepatitis B. Pegylated IFN-α is effective in achieving a sustained virological response, which is defined by hepatitis e antigen (HBeAg) seroconversion and/or HBV DNA levels below 20,000 copies/ml at 6 months after completion of the therapy, in about 30% of HBeAg-positive and 40% of HBeAg-negative cases [25]–[27]. The antiviral mechanisms of the cytokine and the reasons for the differential therapeutic response among the treated patients remain to be elucidated.

In order to investigate the role of TRIM proteins in HBV replication, host innate antiviral response and pathogenesis, 38 human TRIMs were tested in a human hepatocyte-derived cell line for their ability to modulate HBV replication. The study revealed that ectopic expression of 8 TRIMs significantly suppressed HBV transcription. In particular, TRIM41 specifically inhibited the transcription of HBV, which depended on TRIM41 E3 ubiquitin ligase activity as well as the integrity of TRIM41 C-terminal region, but it did not inhibit immediate early promoter activity of cytomegalovirus. Moreover, endogenous TRIM41 plays a role in regulation of HBV transcription in human hepatoma cells. Our study has thus identified TRIMs that inhibit HBV replication and supported the notion that TRIM family proteins serve as innate immune components to shape viral pathogenesis.

## Results

### Identification of TRIM Proteins that Modulate HBV Replication in Human Hepatocyte-derived Cells

**Figure 1:**
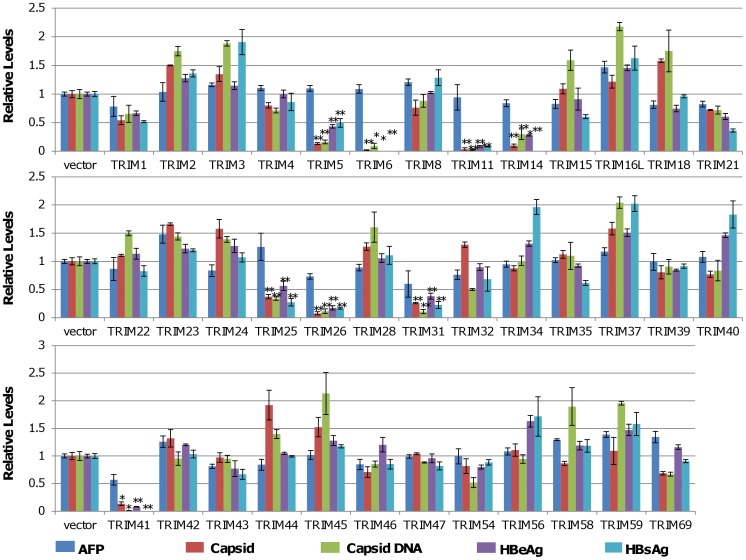
Identification of TRIMs that regulate HBV viral protein expression, capsid assembly and DNA replication. HepG2 cells in 12 well plates were co-transfected with 0.4 µg of pHBV1.3 and 0.4 µg of plasmid expressing an indicated TRIM protein or a control vector. 72 hours after transfection, the levels of the secreted HBeAg and HBsAg were determined by ELISA. The amounts of intracellular capsid and capsid DNA were determined by a viral particle gel assay. The levels of AFP in cultured media were determined by ELISA and served as a cellular function and viability control. The levels of HBeAg, HBsAg, capsid, capsid DNA and AFP from the samples co-transfected with pHBV1.3 and an indicated TRIM-expressing plasmid were expressed as a ratio over that obtained from samples co-transfected with pHBV1.3 and vector plasmids. The mean and standard deviations (n = 3) were presented. * and ** indicate P<0.05 and 0.01, respectively.

In order to systematically investigate the role of TRIM proteins in HBV replication and innate antiviral immune response, 38 plasmids expressing individual TRIMs were purchased from the indicated commercial sources (Table S1). To evaluate the effects of ectopic expression of individual TRIMs on HBV replication in human hepatocytes, HepG2 cells were co-transfected with a plasmid containing 1.3 fold unit-length of HBV genomic DNA (pHBV1.3) and a plasmid expressing a specific TRIM or vector plasmid (pcDNA3.1). The culture media and cells were harvested three days post transfection. Secreted HBV surface antigen (HBsAg) and e antigen (HBeAg) in culture media were quantified by ELISA. The amounts of intracellular capsid and capsid DNA intermediates were measured by a native agarose gel-based particle gel assay [28]. The amount of secreted alpha fetal protein (AFP) was determined by ELISA and served as a control for hepatoma cell-specific gene expression. Viability of the transfected cells was determined by using a CCK-8 kit. As shown in Fig. 1, while ectopic expression of any of the 38 individual TRIMs in HepG2 cells did not apparently affect the cell viability (Fig. S1) and secretion of AFP was only modestly reduced in cells transfected with plasmids expressing TRIM 26, 31 or 41 (Fig. 1), expression of TRIM5, 6, 11, 14, 25, 26, 31 or 41 reduced the amounts of secreted HBsAg and HBeAg by more than 2 folds. Moreover, the particle-gel assay revealed that the same eight TRIMs also significantly reduced the amounts of intracellular capsid and capsid DNA. Interestingly, in contradiction to a previous report which showed that TRIM22 inhibited HBV gene transcription in human hepatoma cells [11], our study did not reveal any significant effect of TRIM22 on HBV protein expression and DNA replication (Fig. 1), which is, in fact, consistent with a recent report by Mao and colleagues [30]. Proper expression of TRIM22 and other eight active TRIM proteins had been confirmed by quantitative RT-PCR assays and/or Western blot analyses (Fig. S2). The simultaneous reduction of the multiple HBV gene products in cells ectopically expressing each of the eight TRIMs implies that these TRIM proteins most likely suppressed viral RNA transcription and/or promoted viral RNA decay. Alternatively, it is also possible that some of the TRIMs may selectively inhibited viral mRNA translation.

### Eight TRIM Proteins Efficiently Reduced the Amount of HBV mRNA

**Figure 2:**
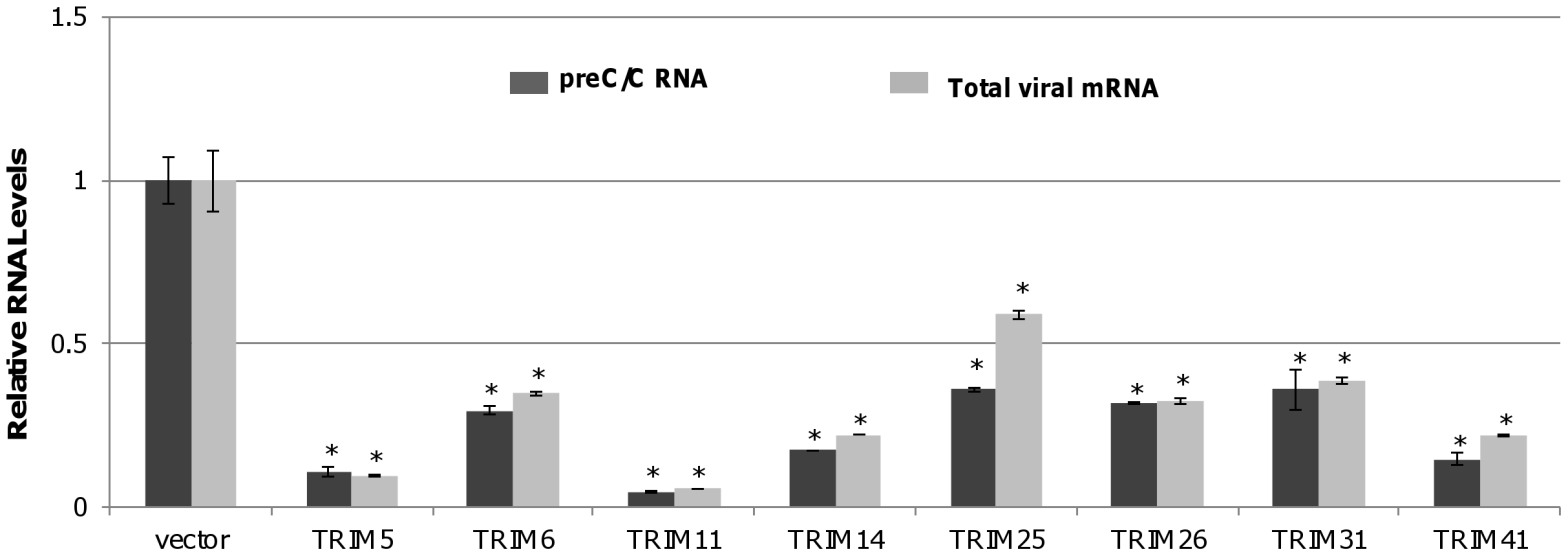
Eight TRIM proteins can efficiently reduce the amount of HBV mRNA in HepG2 cells. HepG2 cells in 12 well plates were co-transfected with 0.4 µg of pHBV1.3 and 0.4 µg of vector plasmid or plasmid expressing one of eight TRIM proteins. Two days post transfection, cells were harvested and total cellular RNAs were extracted. The levels of preC/C RNA and total viral mRNA were quantified by a quantitative RT-PCR assay. The mean and standard deviations (n = 3) were presented. * and ** indicate P<0.05 and 0.01, respectively.

To rule out the possibility that these TRIMs selectively inhibited viral mRNA translation, HBV preC/C RNA and total mRNA were quantified in HepG2 cells co-transfected with pHBV1.3 and a plasmid expressing one of the eight active TRIMs or vector plasmid (pcDNA3.1). As shown in Fig. 2, all the eight TRIMs significantly reduced the amounts of intracellular viral preC/C RNA and total viral mRNA, suggesting that these TRIMs most likely inhibit HBV transcription and/or promote the decay of the viral RNA.

### All of the Eight TRIMs Efficiently Inhibited the Transcriptional Activity of HBV Enhancer I and Enhancer II/core Promoter

**Figure 3:**
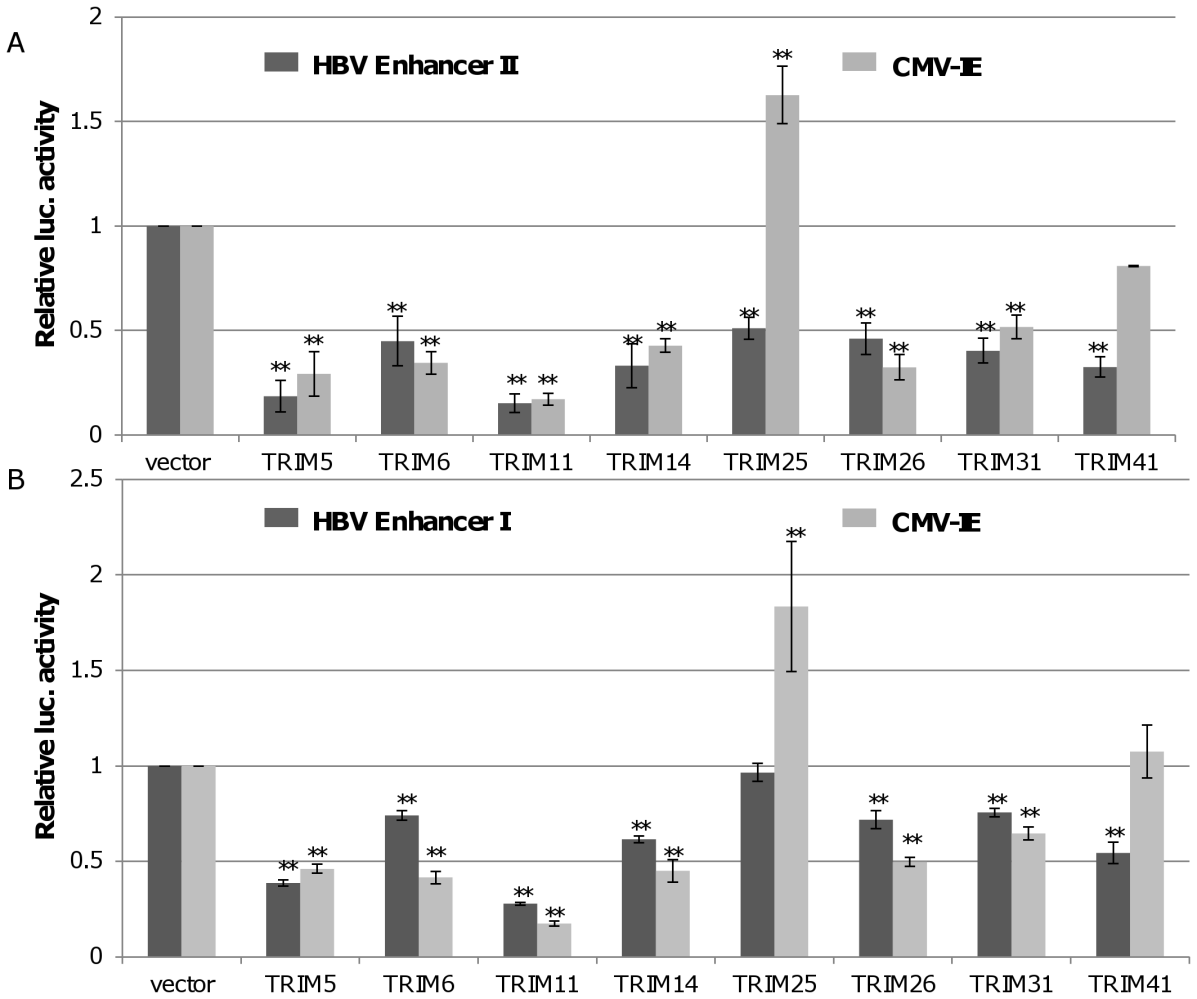
Effects of the eight TRIM proteins on HBV Enhancer I and Enhancer II activity in HepG2 cells. 0.2 µg of pGL4.10-HBV Enh II or pGL4.10-HBV Enh I was co-transfected into HepG2 cells in 24 well plates with 0.2 µg of a vector plasmid or a plasmid expressing an indicated TRIM protein, with 0.1 µg of pCMV-renilla luciferase as an internal control. After two days post transfection, cells were harvested and the luciferase activity was analyzed with a dual-luciferase kit. The mean and standard deviations (n = 4) were presented. * and ** indicate P<0.05 and 0.01, respectively.

HBV transcribes four groups of viral RNA, including 3.5 kb pre-core/core RNAs, 2.4 kb RNA for large envelope protein (L) and 2.1 kb RNAs for middle (M) and small (S) envelope protein and 0.7 kb RNA for X protein, under the control of four promoters and two enhancers, enhancers I and II (Enh I and Enh II) [31], [32]. To examine whether the TRIM-induced reduction of viral RNAs is due to the inhibition of viral gene transcription, effects of the eight TRIMs on HBV Enh I and Enh II/core promoter (Enh II/Cp) activity were determined by reporter assays in HepG2 cells. Briefly, pGL-4.10 HBV Enh I or Enh II/Cp driven firefly luciferase and pCMV-IE renilla luciferase were co-transfected with a plasmid expressing one of the eight TRIMs or vector control into HepG2 cells. As shown in Fig. 3, expression of TRIMs 5, 6, 11, 14, 26, or 31 efficiently inhibited the transcriptional activity of HBV Enh I and II, as well as CMV IE promoter. On the contrary, expression of TRIM25 only inhibited HBV Enh II/Cp, but not HBV Enh I and CMV IE promoter. Interestingly, TRIM41 significantly suppressed both HBV Enh I and Enh II/Cp without obvious inhibition of CMV IE promoter activity. Due to its strong and selective inhibition on HBV transcription, our subsequent studies were focused on TRIM41.

### Inhibition of Transcription is the Primary Effect of TRIM41 in HBV Genome Replication

**Figure 4:**
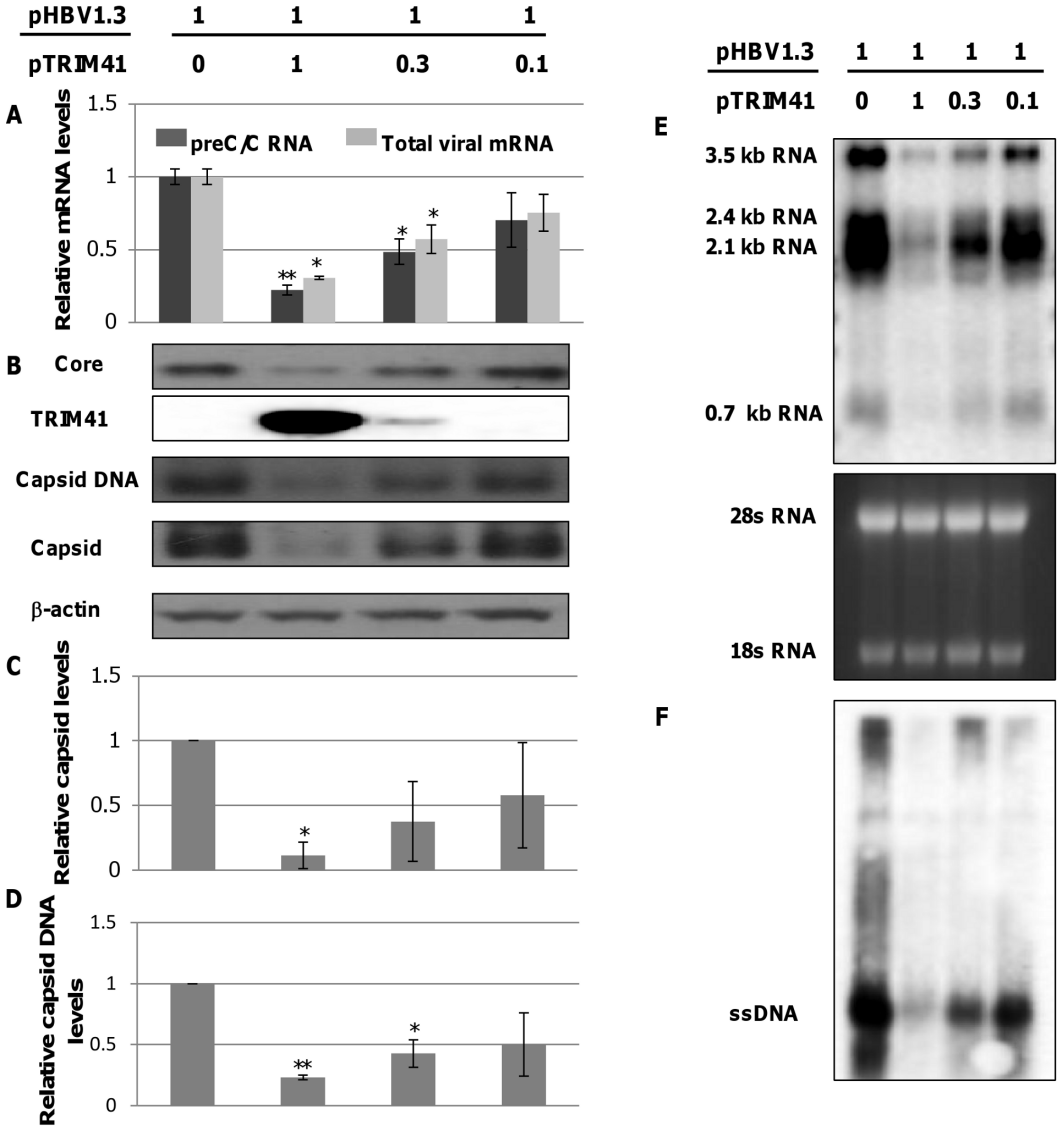
TRIM41 inhibits HBV replication by reducing the HBV mRNA levels. HepG2 cells in 12 well plates were co-transfected with 0.4 µg of pHBV1.3 and the indicated amounts of plasmid expressing TRIM41, supplemented with vector plasmid to make a total of 0.8 µg plasmid. (A) Cells were harvested at two days post transfection. The levels of HBV preC/C RNA and total mRNA were quantified by a real-time RT-PCR assay. (B) The amount of intracellular core protein was determined by a Western blot assay. The level of β-actin was used as a loading control. (C and D) The amounts of intracellular capsid (C) and capsid DNA (D) were determined by a viral particle gel assay. (E) HBV RNAs were determined by Northern blot hybridization. The positions of HBV preC/C RNA (3.5 kb), surface RNAs (sRNA; 2.4 kb and 2.1 kb) and HBx RNA (0.7 kb) are indicated. Ribosomal RNAs (28S and 18S) served as loading controls. (F) The cytoplasmic HBV capsid DNA was detected by Southern blot hybridization. The position of viral single strand DNA (ssDNA) is indicated. The mean and standard deviations were presented (n = 3). * and ** indicate P<0.05 and 0.01, respectively.

In order to further corroborate the effect of TRIM41 on HBV replication with its inhibition of viral RNA transcription, a dose dependent experiment was performed. Briefly, as shown in Fig. 4, fixed amount of pHBV1.3 plasmid was co-transfected with control vector or the indicated amounts of the plasmid expressing TRIM41. Transfected cells were harvested three days post transfection. The amounts of the HBV preC/C RNAs and total viral mRNA were determined by a quantitative RT-PCR assay (Fig. 4A) and all four species of HBV mRNA were also detected by Northern blot hybridization (Fig. 4E). The intracellular core protein was quantified with an immunoblot assay. The capsid and capsid DNA levels were determined by a particle gel assay and/or Southern blot hybridization (Fig. 4B & F). Consistent with the results presented in Figs. 1 and 2, TRIM41 dose-dependently reduced the amounts of viral RNAs. Interestingly, the amounts of viral core protein, capsid and capsid DNA were also proportionally decreased with viral RNAs, implying that the changes of other viral products (or replication intermediates) are the consequence of viral RNA reduction. Hence, the results support the notion that inhibition of HBV transcription is the primary antiviral effect of TRIM41.

### Identification of cis Elements and Transcription Factors Essential for TRIM41 to Inhibit Enh II/Cp Transcription Activity

**Figure 5:**
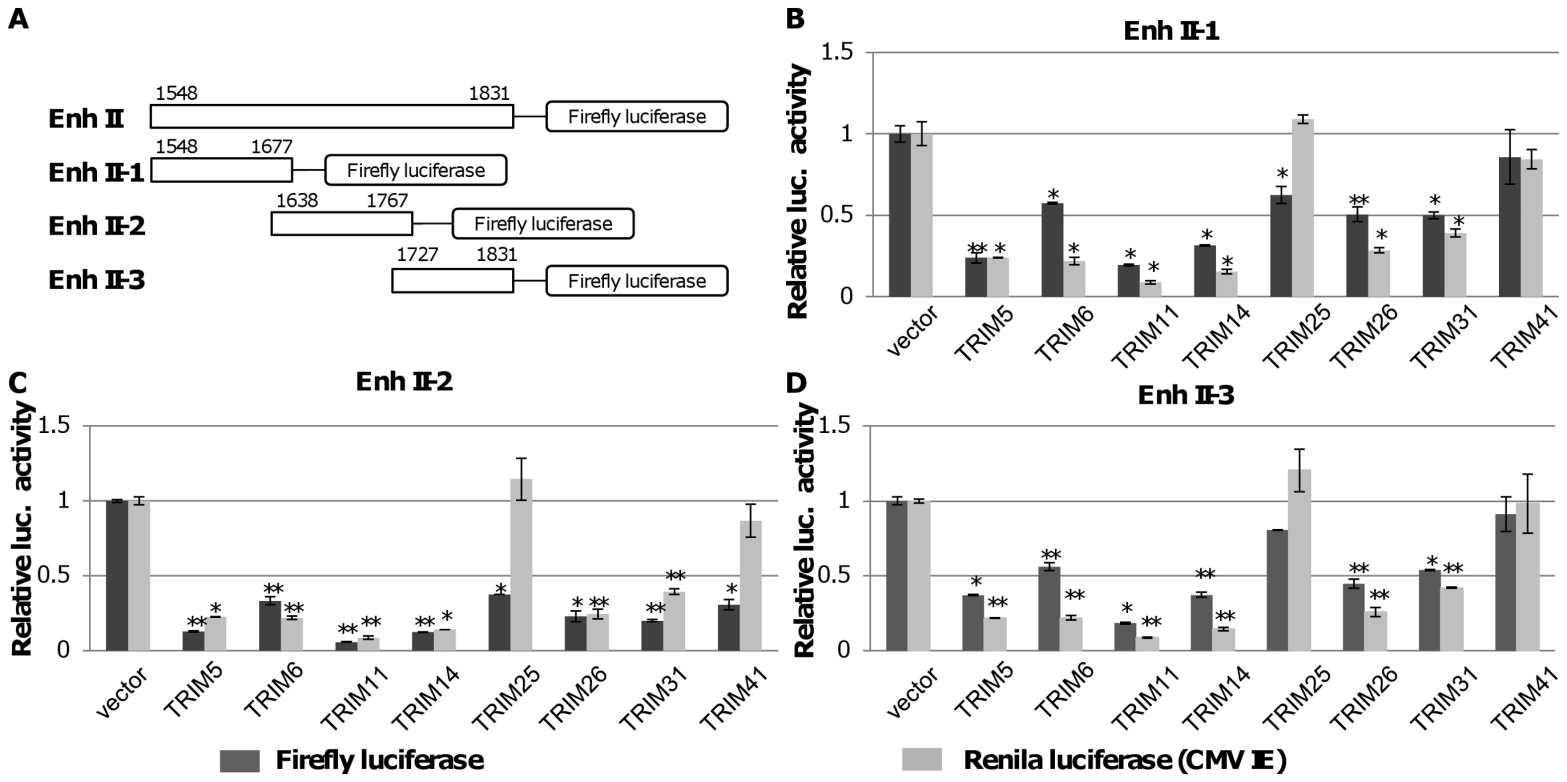
Mapping the *cis* element that mediates TRIMs inhibition of HBV Enh II activity. (A) Schematic representation to map the *cis* element mediating TRIMs inhibition of HBV Enh II activity. Three overlapping DNA fragments covering the entire HBV Enh II region were placed in the up-stream of a firefly luciferase coding sequence to yield three reporter plasmids, pGL4.10-HBV Enh II-1, pGL4.10-HBV Enh II-2 and pGL4.10-HBV Enh II-3, respectively. (B to D) 0.2 µg of the indicated reporter plasmid was co-transfected into HepG2 cells in 24 well plates with 0.2 µg of a vector plasmid or a plasmid expressing the indicated TRIM proteins, with 0.1 µg of pCMV-renilla luciferase as an internal control. Two days post transfection, cells were harvested and the luciferase activity was analyzed with a dual-luciferase kit. The mean and standard deviations (n = 4) were presented. * and ** indicate P<0.05 and 0.01, respectively.

In order to understand how TRIM41 inhibits HBV Enh II/Cp activity, the Enh II/Cp sequence was divided into three fragments (Fig. 5A) and cloned into pGL4.10 to study the expression of firefly luciferase. The effects of TRIM41 and other seven TRIMs on the transcriptional activity of each of the three fragments were tested in the same way as that for full-length Enh II/Cp. As shown in Fig. 5, while TRIM 5, 6, 11, 14, 25, 26, and 31 significantly suppressed the transcriptional activities of all three fragments, TRIM41 specifically inhibited the transcriptional activity of HBV Enh II/Cp Fragment 2. The results thus imply that TRIM41 either directly interacts with the *cis*–elements within the DNA fragment spanning nucleotides 1638 to 1763 or modulate the abundance and/or function of transcription factors bound to this region.

Several transcription factors, such as HNF3-β, RXR-α and PPAR-α, were shown be critical in regulating the activity of the HBV Enh II/Cp fragment 2 [33]–[35]. Hence, the observed inhibition of HBV Enh II/Cp by TRIM41 may be the consequence of TRIM41-induced alteration of abundance and/or function of these three transcription factors. Indeed, as shown in Fig. S3A, ectopic expression of each of the three transcription factors modulated the transcription activity of Enh II/Cp fragment 2. Consistent with the previous observation that HNF3-β inhibits HBV transcription in a transgenic mice model *in vivo* [36], over-expression of HNF3-β significantly inhibited the transcriptional activity of Enh II/Cp fragment 2(Fig. S3A). On the contrary, expression of either RXR-α or PPAR-α significantly enhanced the luciferase expression driven by Enh II/Cp Fragment 2 (Fig. S3A). Interestingly, expression of TRIM41 completely attenuated the activation of transcriptional activity of Enh II/Cp Fragment 2 resulting from ectopically expressed RXR-α or PPAR-α. To obtain additional mechanistic insight, we determined the effects of TRIM41 on the abundance of RXR-α or PPAR-α. The results shown in Fig. S3B demonstrated that expression of TRIM41 did not reduce the amounts of the two transcription factors. Hence, the results presented herein indicate that TRIM41 inhibition of HBV Enh II/Cp transcription is most likely not through down-regulation of the levels of transcription factors RXR-α or PPAR-α in hepatocytes.

### The E3 Ubiquitin Ligase Activity of TRIM41 is Required for Inhibition of HBV Transcription

**Figure 6:**
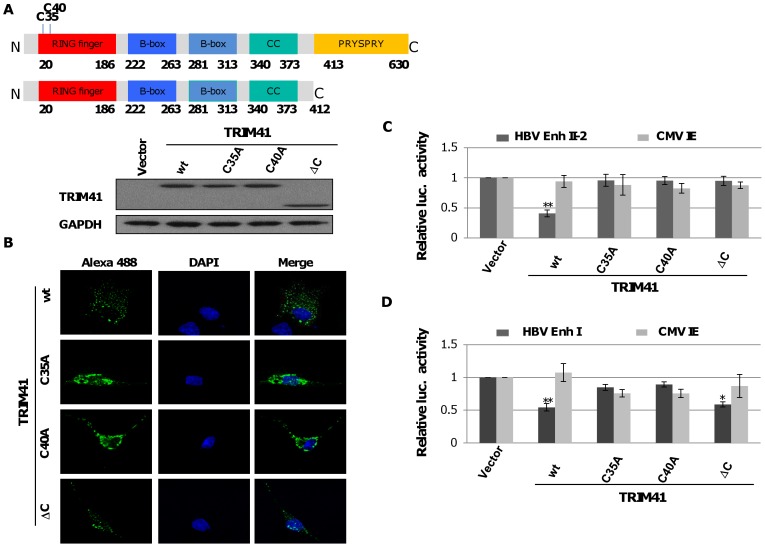
RING finger and PRYSPRY domains of TRIM41 are essential for the inhibition of HBV Enh II activity. 0.4 µg of plasmids expressing wild type or mutant TRIM41 were transfected into HepG2 in 24 well plates. The cells were harvested at two days post transfection. Expression of TRIM41 was determined by Western blot assays (A) or immunofluorescence (B). (C) 0.2 µg of pGL4.10-HBV Enh II was co-transfected into HepG2 cells in 24 well plates with 0.2 µg of a vector plasmid or plasmid expressing the wild type or indicated mutant TRIM41, with 0.1 µg of pCMV-renilla luciferase as an internal control. Two days post transfection, cells were harvested and the luciferase activity was analyzed with a dual-luciferase kit. The mean and standard deviations (n = 4) were presented. * and ** indicate P<0.05 and 0.01, respectively.

The N-terminus of TRIM41 is comprised of three conserved domains, including a RING finger domain, a B box domain, and a coiled coil domain. The C-terminus contains a PRY-SPRY domain. In order to determine whether the E3 ligase activity is essential for TRIM41 to inhibit HBV transcription, two key cysteine residues 35 and 40 in the RING finger domain essential for the E3 ubiquitin ligase activity were mutated to alanine, which yielded mutants TRIM41C35A and TRIM41C40A, respectively [37]–[39]. In addition, a TRIM mutant with a deletion of the entire C-terminal PRY-SPRY domain was constructed and designated as TRIM41ΔC. As shown in Fig 6, expression of the three TRIM41 mutants could be readily detected by Western blot assay in the lysates of transfected cells (Fig. 6A) and by immunofluorescent analysis in transfected cells (Fig. 6B). TRIM41 wild-type and mutants predominantly localized in the cytoplasm. However, in agreement with the results presented above, while wild-type TRIM41 efficiently inhibited both Enh I and Enh II/Cp activities, TRIM41C35A and TRIM41C40A did not affect the transcriptional activities of neither HBV enhancers (Figs. 6C and D). The results thus suggest that the putative ubiquitin E3 ligase activity is essential for TRIM41 to regulate HBV transcription. However, it was very interesting to observe that TRIM41ΔC significantly inhibited the transcriptional activity of Enh I, but not Enh II (Figs. 6C and D), suggesting that the integrity of the C-terminal PRY-SPRY domain is essential for TRIM41 to suppress Enh II transcription, but dispensable for regulation of Enh I.

### Endogenous TRIM41 in HepG2.2.15 Cells Suppresses HBV Transcription

**Figure 7:**
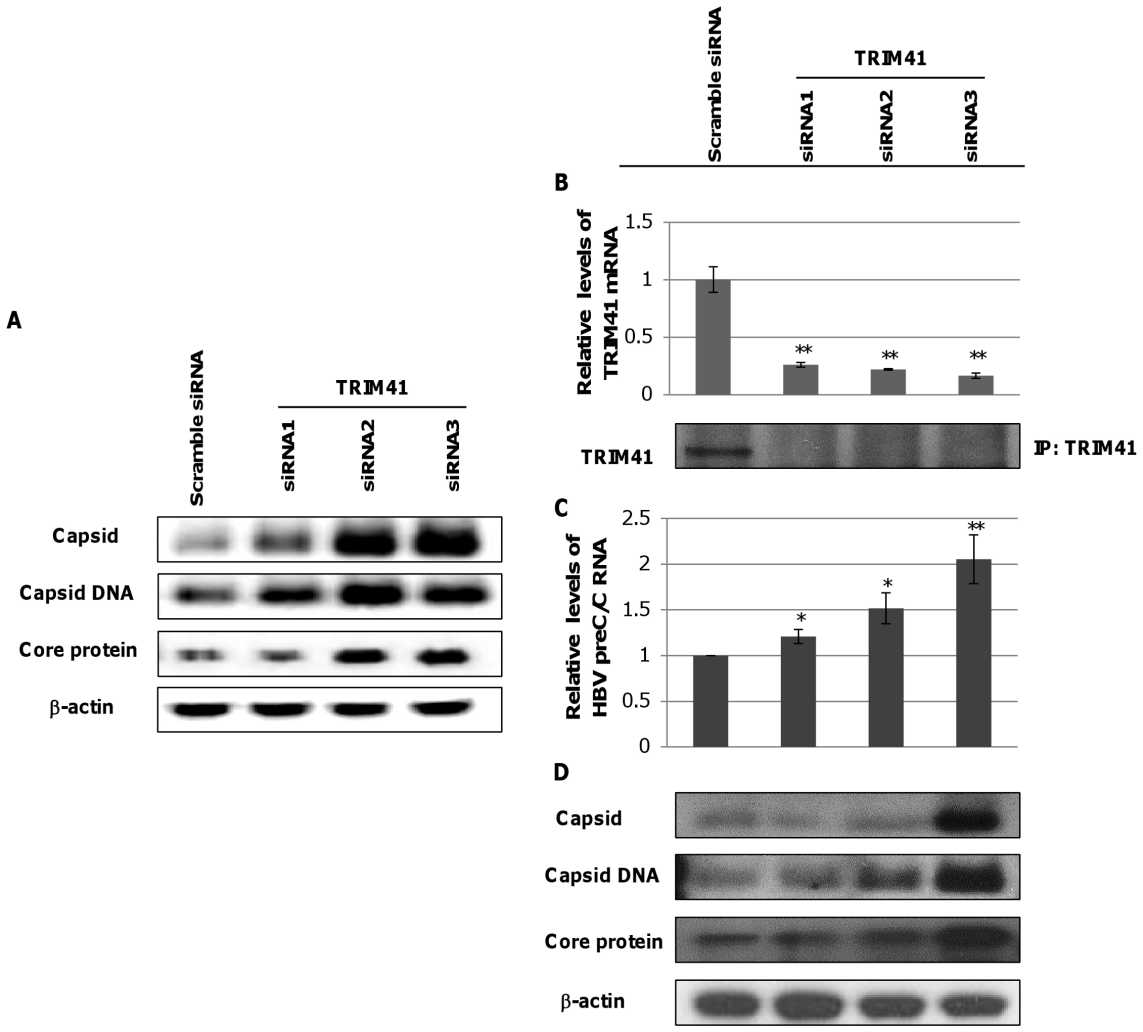
Endogenous TRIM41 inhibits HBV transcription in HepG2 cells. (A) HepG2 cells in 12 well plates were transfected with 20 nM of scramble siRNA or one of three siRNA specifically targeting TRIM41. Six hours later, the cells were co-transfected with 0.4 µg of pHBV1.3 and 0.4 µg of a plasmid expressing TRIM41. The cells were re-transfected with 20 nM of siRNA 24 hours later and harvested at 48 hours post pHBV1.3 and TRIM41-expressing plasmid transfection. Core protein was tested by Western blot with β-actin as a loading control. Capsid and capsid DNA were analyzed by a particle gel assay. (B) 15 nM of TRIM41 siRNAs or scramble siRNA were transfected into HepG2.2.15. Two days post transfection, cells were harvested. The total cellular RNAs were extracted and reverse transcribed. TRIM41 siRNA knockdown efficiency was tested by quantification of TRIM41 mRNA level with a quantitative RT-PCR assay (upper panel). The level of TRIM41 protein in the transfected cultures were determined by immunoprecipitation and Western blot assays with a polyclonal antibody against human TRIM41 (lower panel). (C) HBV preC/C RNA and total viral mRNA were quantified by a quantitative RT-PCR assay and normalized with β-actin mRNA as an internal control. (D) Core protein, capsid and capsid DNA levels were tested by Western blot or Southern blot assays. The same amounts of cell lysates were loaded after BCA quantification. The level of β-actin was used as a loading control. The mean and standard deviations (n = 4) were presented. * and ** indicate P<0.05 and 0.01, respectively.

Aforementioned transcriptional regulatory effects of TRIM41 on HBV were demonstrated *via* ectopic expression of the protein in hepatoma cells. In agreement with these results, we further demonstrated that reducing the ectopically expressed TRIM41 by small interfering RNA (siRNA) in the HepG2 cells co-transfected with pHBV1.3 and plasmid expressing TRIM41 significantly increased the amounts of HBV core protein, capsid and capsid DNA (Fig. 7A). To substantiate the pathobiological function of TRIM41 in HBV infection, we tested if endogenous TRIM41 played a role in modulating viral replication. TRIM41 expression was knocked down by siRNA in HepG2.2.15 cells that harbored an integrated head-to-tail dimmer of HBV genome and supported stable HBV replication [40]. As shown in Fig. 7B, each of the three siRNAs targeting TRIM41 efficiently reduced the amounts of TRIM41 mRNA and protein in HepG 2.2.15 cells, albeit at a different efficiency. Interestingly, the increase in HBV preC/C mRNA was observed to positively correlate with the extent of TRIM41 reduction in siRNA transfected cells (Fig. 7C). As a consequence, the amounts of viral core protein, capsid and capsid DNA also proportionally increased in the cells where TRIM41 level was down-regulated (Fig. 7D). These results thus strongly suggest that endogenously expressed TRIM41 in hepatoma cells indeed suppresses HBV replication.

## Discussion

Due to the lack of convenient cell culture systems supporting efficient HBV infection, HBV replication is mainly investigated in human hepatoma cells transfected with HBV genomes. Obviously, only the post entry events of HBV replication can be studied in transfected cells. Using the HBV genome transiently transfected cell culture system, we identified eight members of TRIM family proteins that significantly reduced all the viral gene products, including viral mRNA, protein, capsid and capsid DNA, upon ectopic expression in HepG2 cells (Figs. 1 and 2). Furthermore, all the eight TRIMs were demonstrated to be able to inhibit the transcriptional activities of HBV Enh I and/or Enh II/Cp in HepG2 cells (Fig. 3).

However, based on their differential effects on HBV Enh I, Enh II/Cp and CMV IE promoter, the eight TRIMs can be categorized into two groups. Group I includes six TRIMs: TRIM 5, 6, 11, 14, 26 and 31. Each TRIM inhibited the HBV Enh I, Enh II/Cp and CMV IE promoter-driven luciferase expression to a similar extent (Fig. 3). Analysis of both HBV enhancer II and CMV IE promoter indicates that the two transcriptional regulatory DNA fragments share binding sites of several transcription factors, which may mediate the transcriptional suppression of this group of TRIMs in both promoters. Alternatively, these TRIMs may target host’s general transcription regulatory proteins, such as histone modification enzymes, epigenetic readers and transcription co-activators, to alter the transcription of a broad spectrum of host and viral genes [41]. Group II includes TRIM25 and TRIM41, which selectively inhibited HBV enhancers, but not CMV IE promoter (Fig. 3). Specifically, TRIM25 slightly enhanced the transcriptional activity of CMV IE promoter and selectively inhibited the transcriptional activity of HBV Enh II/Cp, but not Enh I. TRIM25 can be induced by estrogen and IFN-α and is involved in diverse cellular functions (Fig. S4) [42], [43]. Estrogen suppresses HBV replication and is a host factor contributing to lower HBV titers in women [44], [45]. Mechanistically, it was demonstrated that estrogen receptor-α squelched HNF4-α binding to HBV enhancer I [46]. Its estrogen inducible expression and HBV transcriptional inhibition activity suggest that TRIM25 may also play a role in estrogen suppression of HBV infection. Moreover, TRIM25 is not only an IFN-stimulatedgene, but also an essential component of RLR-driven IFN response [16]. Therefore, TRIM25 may play an important role in restricting HBV infection through both direct inhibition of HBV transcription and induction of RLR-driven antiviral responses [47]. However, although ectopic expression of TRIM25 inhibited HBV Enh II/Cp activity and restricted the viral replication, knockdown of endogenous TRIM25 in HepG2.2.15 cells did not apparently alter the abundance of HBV mRNA and other viral replication intermediates (data not shown). TRIM41 has been reported to localize in the cytoplasm and nucleus, and it can induce the degradation PKC-α through proteasome pathway [37], [38]. In this study, we demonstrated that it did not alter the transcriptional activity of CMV IE promoter, but efficiently suppressed the transcriptional activities of both Enh I and Enh II/Cp. Mechanistic analyses further indicated that a HBV DNA segment spanning nt 1638 to 1763 was essential for TRIM41 to inhibit HBV enhancer II activity and the inhibitory effect depended on TRIM41 E3 ubiquitin ligase activity as well as the integrity of TRIM41 C-terminal region. However, the C-terminal region of TRIM41 was not essential for inhibition of HBV Enh I activity, suggesting that the protein regulates HBV Enh I and II *via* distinct mechanisms. Knockdown of endogenous TRIM41 expression in a HepG2.2.15 cells significantly increased the levels of HBV preC/C RNA and consequentially, the viral core protein, capsid and capsid DNA were also increased. These results thus unambiguously demonstrated that endogenous TRIM41 plays a role in regulation of HBV transcription in human hepatoma cells.

Inflammatory cytokines are important mediators of host innate and adaptive immune responses and are essential for the resolution of HBV infection [48]–[54]. Particularly, IFNs are the primary antiviral cytokines that inhibit HBV transcription and reduce the amount of pgRNA-containing nucleocapsids [30], [55], [56]. Induction of TRIM5, 6, 25, 26 and 31 expression by IFN-α and/or IFN-γ in human hepatocytes may partially contribute to the suppression of HBV transcription by the cytokines (Fig. S4 and S5). In addition, two independent studies showed that IFN-induced reduction of pgRNA-containing nucleocapsids required proteasome activity [57], [58], suggesting that ubiquitination and proteasome-mediated cellular processes may be involved in the antiviral activity of IFNs. However, among the 38 TRIMs tested, none of TRIMs reduced the amount of viral DNA without reducing the amount of viral mRNA, suggesting that none of the tested TRIMs directly targets HBV nucleocapsid assembly process or stability. In addition to IFNs, TNF-α also inhibits both HBV Enh II/Cp and CMV IE promoter activities in hepatoma cells (Fig. S5). Interestingly, TNF-α can induce TRIM31 expression in both HepG2 and Huh7 cells. It is thus possible that TRIM31 may play a role in TNF-α induced suppression of HBV and CMV transcription. Ironically, IL-1β is a more potent TRIM31 inducer than TNF-α, but is a weaker inhibitor of HBV Enh II/Cp and modest stimulator of CMV promoter in HepG2 and Huh7 cells (Fig. S4 and S5). The apparent contradictive observations imply that the biological consequence of a given TRIM protein’s function is circumstantially modulated in different cellular processes.

In conclusion, through a systematic screen of 38 human TRIM proteins, we identified 8 TRIMs that efficiently suppressed HBV transcription. They may play a role in modulating HBV replication under certain pathophysiological conditions. Particularly, we provide strong evidences, which suggest that endogenously expressed TRIM41 modulates HBV gene expression in human hepatocyte-derived cells and thus may play a role in HBV pathogenesis *in vivo.*

## Materials and Methods

### Cell Culture

The human hepatoma derived cell lines HepG2 and Huh7 (Purchased from ATCC) were cultured in DMEM/F12 (Invitrogen) supplemented with 10% fetal bovine serum (Invitrogen), 2 mM L-glutamine, 100 U/ml penicillin, and 100 µg/ml streptomycin at 37°C under humidified air containing 5% CO_2_. As for HepG2.2.15 (Purchased from Aybio Shanghai, China), 300 µg/ml geneticin (Invitrogen), 1% DMSO was added to DMEM/F12 (Gibco) supplemented with 10% fetal bovine serum (Invitrogen), 2 mM L-glutamine, 100 U/ml pencillin, and 100 µg/ml streptomycin.

### Plasmid Construction

pHBV1.3, which is kindly provided by Dr. Qiang Deng in Pasteur Institute of Shanghai, contains 1.3-fold over length HBV genomic sequence. HNF3-β was cloned into pcDNA3.1(+). HBV Enh I (spanning nucleotide 686 to nucleotide 1359 of HBV genome), HBV Enh II (spanning nucleotide 1548 to nucleotide 1831 of HBV genome), HBV Enh II-1(spanning nucleotide 1548 to nucleotide 1677 of HBV genome), HBV Enh II-2(spanning nucleotide 1638 to nucleotide 1767 of HBV genome) and HBV Enh II-3(spanning nucleotide 1727 to nucleotide 1831 of HBV genome) were cloned into pGL-4.10 after digestion with KpnI and XhoI. pCMV Renilla luciferase was purchased from Genescript, and the renilla luciferase was cloned into pGL4.10 basic with CMV as its promoter. Information for plasmids expressing individual TRIM proteins is provided in Table S1.

### Transient Transfection and RNA Interference

One day before transfection, cells were trypsinized and seeded onto plates, 1×10^5^/well in 12 well plates or 0.5×10^5^/well in 24 well plates. Before transfection, the media in the plates were changed with those without antibiotics. In plasmid transfection, X-TREMEGENE HP DNA transfection reagent (Roche) was used, while in siRNA transfection, Lipofectamine RNAiMAX (Invitrogen) was used. One day after transfection, the media were changed those containing antibiotics. TRIM41 siRNAs were purchased from Ambion, and a final concentration of 15 nM was used for the transfection of HepG2.2.15.

### ELISA

Culture medium was diluted 1∶30 for HBeAg or 1∶3 HBsAg in PBS (Gibco) and the levels of HBeAg or HBsAg were measured by using the HBeAg or HBsAg ELISA kit (Bluegene) according to the manufacturer’s direction.

### Cell Viability Assay

Cell viability was determined with cell counting kit-8 (Dojindo) according to the manufacturer’s direction.

### Viral Particle Gel Assay

Cells were lysed in the lysis buffer (50 mM Tris-HCl [pH 7.5], 150 mM NaCl, 0.5% CA-630, supplemented with a protease inhibitor cocktail) at room temperature for 10 minutes, spun down at 13,200 rpm for 10 minutes. The supernatants were collected and treated with DNase I, followed by separation on a 1% agarose gel in 1xTris-acetate-EDTA (TAE) buffer. In order to detect capsid DNA, the agarose gel was first denatured by soaking the gel in denaturation buffer (0.5 M NaOH and 1.5 M NaCl), then neutralized in neutralization buffer (1.5 M NaCl and 1 M Tris-HCl [pH 7.4]), blotted onto positively charged nylon membranes, and hybridized with 1 kb dig-labeled probe. The viral DNAs were detected by hybridization and visualized using Kodak X-film. The probe for capsid DNA detection was prepared by PCR with primers -1252-TCCTCTGCCGATCCATACTG-1271-3′ (forward) and 5′-2095-CCACCCAGGTAGCTAGAGTCA-2115-3′ (reverse).

In order to test viral capsid, the agarose gel was directly blotted onto ECL membranes by capillary blotting. The membranes were blocked with 10% non-fat milk in TBS–0.1% Tween 20 for 30 minutes and were incubated with primary antibodies HBc (Dako) for overnight at 4°C. Membranes were washed thrice with each time 10 minutes with TBS–0.1% Tween 20. The HRP conjugated secondary antibody against rabbit (1∶5000) was added to membrane and incubated for 1 hour at room temperature. Membranes were washed thrice with each time 10 minutes with TBS–0.1% Tween 20. Membrane was incubated with substrate for 5 minutes, and then exposed to Kodak X-film or visualized with Biorad’s ChemiDoc MP instrument.

### Southern and Northern Blot Hybridization

Cells were harvested 72 hours post-transfection and HBV capsid DNA was extracted as previously described [28] and subjected to electrophoresis in 1.5% agarose gel and blotted onto positively charged nylon membranes. HBV DNA was hybridized with 1 kb dig-labeled probe and visualized by AP conjugated anti-dig antibody [29]. For detection of HBV mRNA, total cellular RNAs were extracted with RNeasy Plus Kit (Qiagen). Five micrograms of total RNAs were resolved in 1.3% agarose gel containing 0.66 M formaldehyde and transferred onto positively charged nylon membranes and probed with 7 ng/ml dig labeled 350 nt RNA probe, synthesized by T7 RNA polymerase. The dig signal was detected by AP conjugated anti-dig antibody [29].

### Western Blot Assay

Cells were harvested 48 hours post-transfection or after the indicated treatment time. Cells were washed once with room-temperature PBS and lysed with lysis buffer (50 mM Tris-HCl [pH 7.5], 150 mM NaCl, 0.5% CA-630, supplemented with a protease inhibitor cocktail). Cell lysates were mixed with 4×SDS loading buffer and 10 mM DTT, and were boiled for 5 minutes at 100°C. Equal amounts of proteins were subjected to SDS-polyacrylamide gel electrophoresis (PAGE) and were then transferred to nitrocellulose membranes (Invitrogen). The membranes were blocked with 10% nonfat milk in TBS–0.1% Tween 20 for at least 30 minutes and were then incubated with the indicated primary antibodies against FLAG (Sigma), TRIM41 (Abcam), β-actin (Sigma), HBc (Dako), GAPDH (Cell signaling) for overnight at 4°C. Membranes were washed thrice with each time 10 minutes with TBS–0.1% Tween 20. The HRP conjugated secondary antibody against rabbit (1∶5000) or mouse (1∶5000) was added to membrane and incubated for 1 hour at room temperature. Membranes were washed thrice with each time 10 minutes with TBS–0.1% Tween 20. Membranes were incubated with substrate for 5 minutes, and then exposed to Kodak X-film or visualized with Biorad’s ChemiDoc MP instrument.

### Luciferase Reporter Assay

5×10^4^ cells were seeded into each well of 24 well plates. After incubation for 16 hours, cells were transfected with 0.2 µg of firefly luciferase containing plasmid and 0.1 µg of pCMV Renilla luciferase, as well as the indicated 0.2 µg of TRIM expression plasmid. After transfection for 48 hours, cells were harvested with the addition of 450 µl cell lysis buffer. The luciferase assays were performed according to the manufacturer’s instructions (Promega).

### Quantitative Real-time Polymerase Chain Reaction

Total RNA was isolated from HepG2, Huh7, and HepG2.2.15 cells by RNeasy Plus Kit (Qiagen) and was reverse-transcribed (RT) using a complementary DNA (cDNA) synthesis kit (Biorad) according to the manufacturer’s instructions. Subsequently, cDNA was subjected to quantitative real-time PCR using a Lightcycler480 and SYBR Green system (Roche Diagnostics) following the manufacturer’s protocol with primers specified in Table S2.

### Immunofluorescence

1×10^5^ HepG2 cells were seeded into 12-well plates. After one day, cells were transfected with indicated TRIM41wt or mutants as indicated. One day post transfection, cells were seeded to chamber slides coated with Poly-lysine. One day later, cells were fixed with 3.7% paraformaldehyde for 20 minutes at room temperature, followed by washing with PBS three times. Then, cells were blocked with 2% BSA in 1×TBS containing 0.5% Triton-X-100 for 30 minutes at room temperature. After being washed with 1×TBS containing 0.1% Triton-X-100 three times, TRIM41 antibody (5 µg/ml) was added to cells, and incubated at room temperature for 1 hour, and then washed with 1×TBS containing 0.1% Triton-X-100 three times. Goat anti Rabbit Ig H+L Alex 488 antibody (0.4 µg/ml) was added to cells to incubate at room temperature for 1 hour, followed by three times PBS washing. Finally, cells were incubated with 2 µg/ml DAPI, and washed 6 times with PBS. Add mount medium to cells and cover with a glass slide, and then the cells were visualized with Leica confocal microscope.

### Immunoprecipitation Assay

2×10^5^ HepG2.2.15 cells were seeded into T75 flask. After one day, cells were transfected with indicated TRIM41 siRNA with a final concentration of 15 nM as mentioned above. Two days post transfection, cells were washed thrice with 15 ml PBS (GIBCO), and then lysed with 1 ml IP buffer (Thermo) containing 1× cocktail (Sigma) for 10 minutes on ice. Cell lysates were centrifuged at 13,200 rpm for 10 minutes 4°C, and the supernatants were transferred to a clean 1.5 ml Eppendorf tube. The protein concentrations were quantified by BCA kit (Thermo). To clear the lysates, the same protein amount of cell lysates was added with 20 µl of protein A/G agarose beads (Shuiyuan Biotech) and incubated by rotation at 4°C for 2 hours, and then cell lysates were collected again by centrifugation and transferred to another new tube. For the binding of TRIM41 with antibody, 5 µg TRIM41 antibody (Abcam) was added to each tube of cell lysates and incubated at 4°C overnight. Next day, 20 µl of protein A/G beads were added to each tube of cell lysates with antibody, and incubated for 2 hours at 4°C. To pellet the beads, the tubes were centrifuged by briefly spinning with the highest speed of 8,000 rpm. The beads were washed with 50×volume PBS of packed beads for 5 times. Then 60 µl of 1× SDS loading buffer (Invitrogen) was added to each tube and proteins were denatured by heat at 100°C for 5 minutes. Finally, the protein samples were subjected to polyacrylamide gel electrophoresis and Western blot to determine the expression of TRIM41.

### Statistical Analyses

Student’s t-test available in Microsoft software Excel was used to determine the statistical significance of the data.

## Supporting Information

Figure S1 **Effect of TRIM expressions on cell viability of the HepG2 cells.** 0.1 µg of pHBV1.3 and 0.1 µg of the indicated TRIM expression plasmid were co-transfected into HepG2 cells in 96 well plates. Three days post transfection, cells were harvested and cell viability was determined with CCK-8 kit according to the manufacturer’s direction. The mean and standard deviations (n = 3) were presented. * and ** indicate P<0.05 and 0.01, respectively.
(TIF)

Figure S2 **Determination of the expression of the nine TRIMs.** HepG2 cells were transfected with 0.4 µg of the indicated TRIM expression plasmids in 24 well plates. Two days post transfection, cells were harvested, and expression of the TRIMs was determined by a quantitative RT-PCR (A) or Western blot (B). The mean and standard deviations (n = 3) were presented. * and ** indicate P<0.05 and 0.01, respectively.
(TIF)

Figure S3 **TRIM41 counteracts the enhancing effects of RXR-α and PPAR-α on HBV enhancer II activity in HepG2.** (A). pGL4.10-HBV enhancer II, pCMV-renilla luciferase and indicated plasmids were co-transfected into HepG2 cells. 48 hours after transfection, luciferase activity was analyzed with a Dual-luciferase kit. The expressions of the indicated proteins were tested by Western blot. (B). RXR-α or PPAR-αwere co-transfected with TRIM41 or TRIM41C35A expression plasmid into HepG2 cells. Two days post transfection, the protein levels were determined by Western blot. The mean and standard deviations (n = 4) were presented. * and ** indicate P<0.05 and 0.01, respectively.
(TIF)

Figure S4 **Effects of five representative inflammatory cytokines on the expression of the eight TRIMs that suppress HBV replication in human hepatoma cells.** HepG2 (A) and Huh7 (B) cells were treated with 1000 IU/ml IFN-α, 10 ng/ml IFN-γ, 10 ng/ml IL-1β, 10 ng/ml IL-6, or 10 ng/ml TNF-α for 24 hours. The levels of the indicated TRIM mRNA were determined by a real-time RT-PCR assay. The mean and standard deviations (n = 3) were presented. * and ** indicate P<0.05 and 0.01, respectively.
(TIF)

Figure S5 **Effects of five representative inflammatory cytokines on HBV Enh II activity in human hepatoma cells.** pGL4.10-HBV enhancer II was transfected into HepG2 cells with pCMV-renilla luciferase as an internal control. Six hours later, cells were treated with 1000 IU/ml IFN-α, 10 ng/ml IFN-γ, 10 ng/ml IL-1β, 10 ng/ml IL-6, or 10 ng/ml TNF-α for 48 hours. Luciferase activity was analyzed with a Dual-luciferase kit. The mean and standard deviations (n = 6) were presented. * and ** indicate P<0.05 and 0.01, respectively.
(TIF)

Table S1 TRIM protein expression plasmids.
(DOCX)

Table S2 Sequence of primers for real time PCR assays.
(DOCX)
