# Supplementary figures and images for: Identification and Characterization of Multiple TRIM Proteins That Inhibit Hepatitis B Virus Transcription
Source: PLoS One. 2013 Aug 1;8(8):e70001. doi: 10.1371/journal.pone.0070001 (PMC3731306; doi:10.1371/journal.pone.0070001)

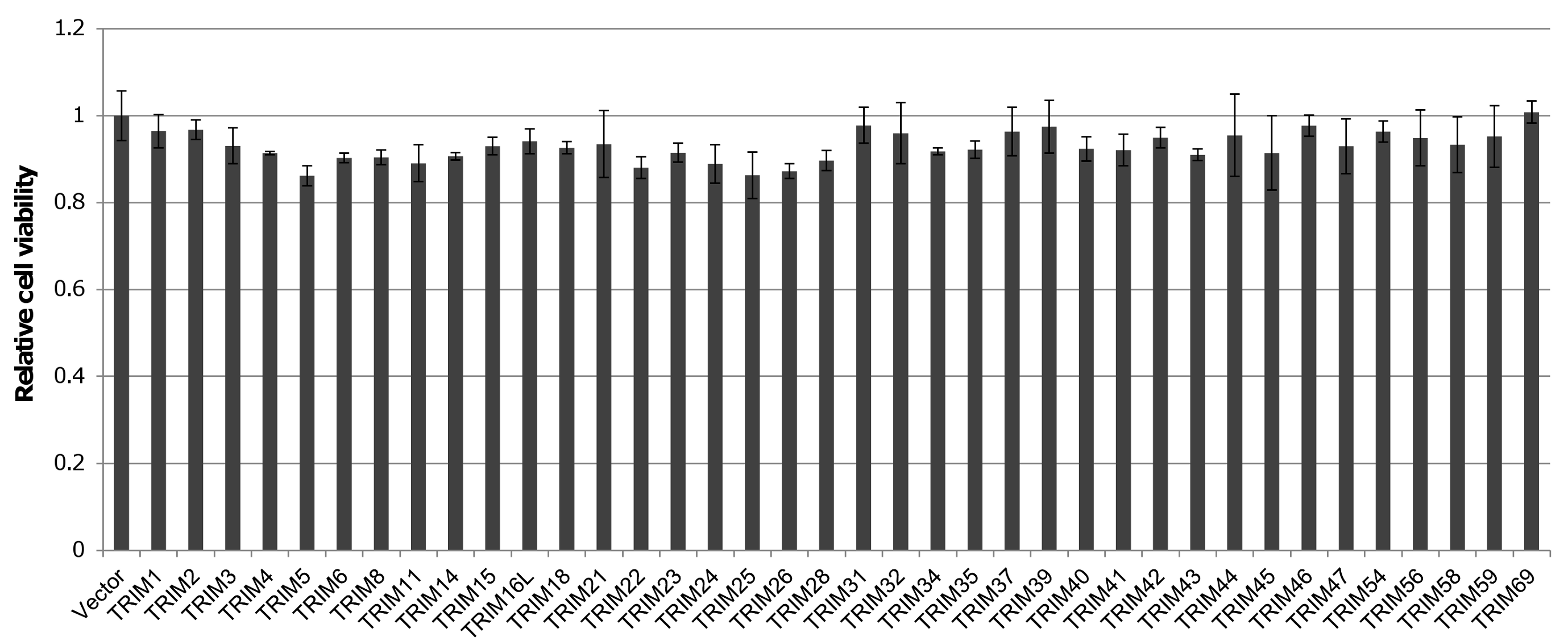

Supplement: Figure S1 — Effect of TRIM expressions on cell viability of the HepG2 cells. 0.1 µg of pHBV1.3 and 0.1 µg of the indicated TRIM expression plasmid were co-transfected into HepG2 cells in 96 well plates. Three days post transfection, cells were harvested and cell viability was determined with CCK-8 kit according to the manufacturer’s direction. The mean and standard deviations (n = 3) were presented. * and ** indicate P<0.05 and 0.01, respectively. (TIF) [file pone.0070001.s001.tif]

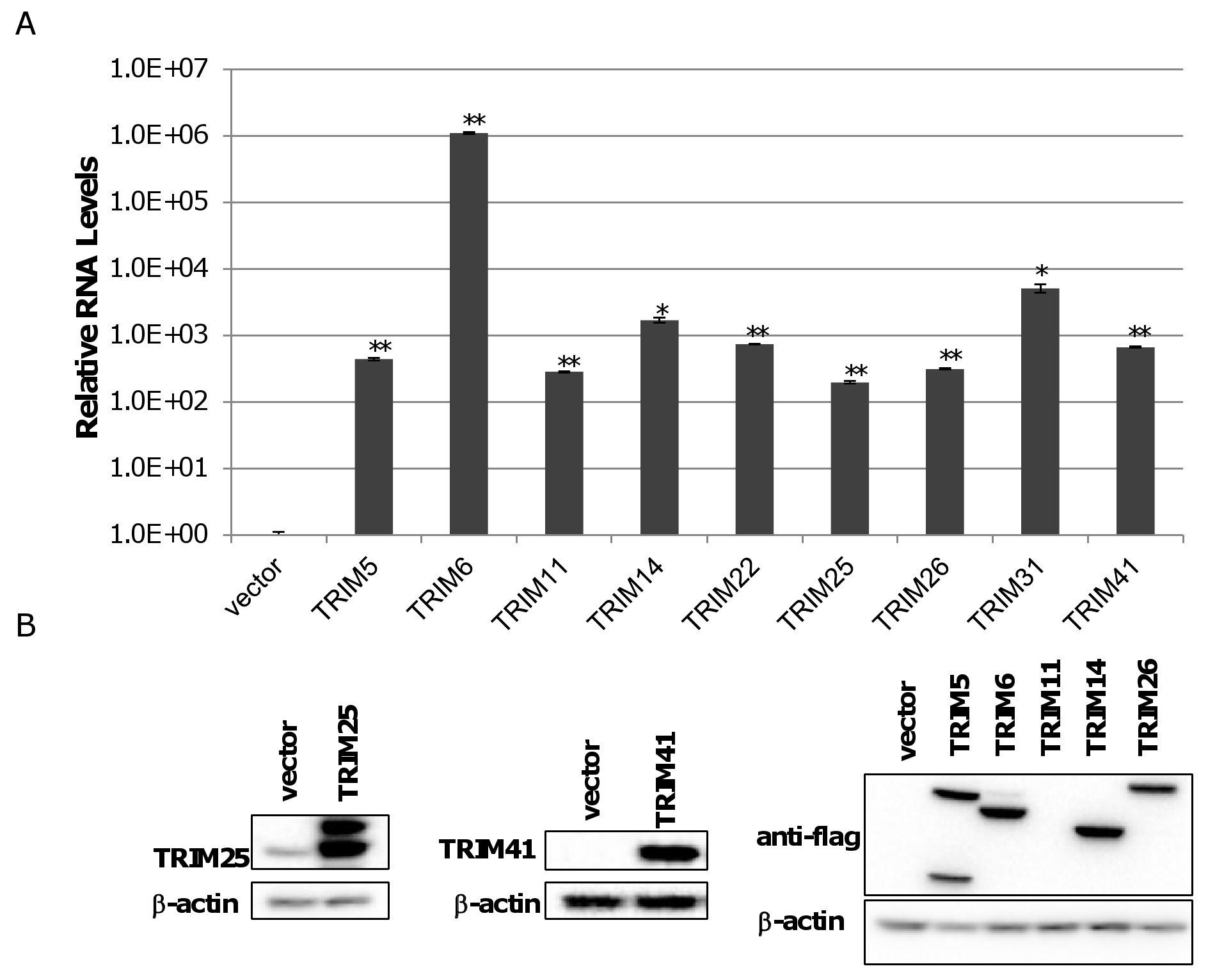

Supplement: Figure S2 — Determination of the expression of the nine TRIMs. HepG2 cells were transfected with 0.4 µg of the indicated TRIM expression plasmids in 24 well plates. Two days post transfection, cells were harvested, and expression of the TRIMs was determined by a quantitative RT-PCR (A) or Western blot (B). The mean and standard deviations (n = 3) were presented. * and ** indicate P<0.05 and 0.01, respectively. (TIF) [file pone.0070001.s002.tif]

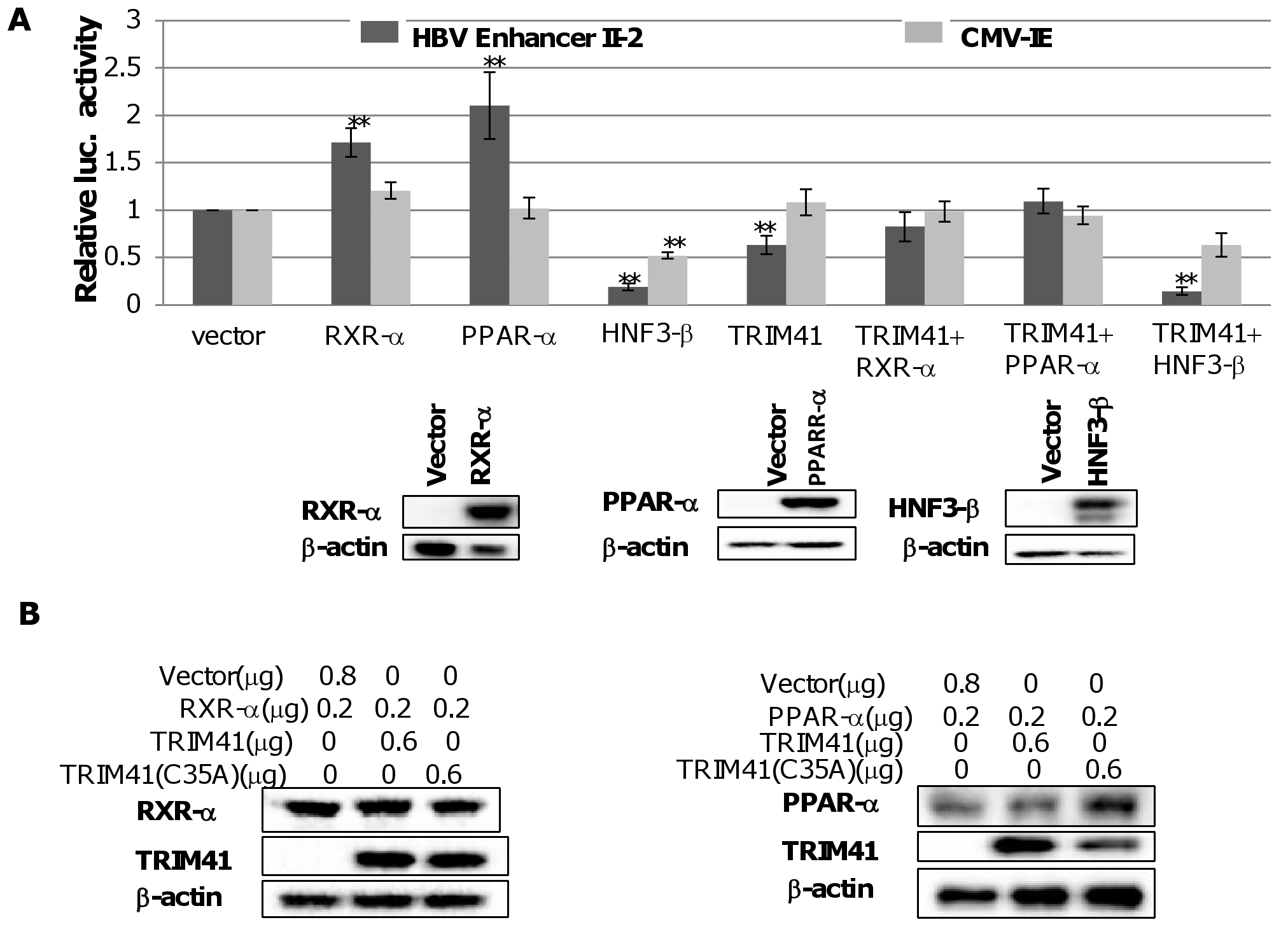

Supplement: Figure S3 — TRIM41 counteracts the enhancing effects of RXR-α and PPAR-α on HBV enhancer II activity in HepG2. (A). pGL4.10-HBV enhancer II, pCMV-renilla luciferase and indicated plasmids were co-transfected into HepG2 cells. 48 hours after transfection, luciferase activity was analyzed with a Dual-luciferase kit. The expressions of the indicated proteins were tested by Western blot. (B). RXR-α or PPAR-αwere co-transfected with TRIM41 or TRIM41C35A expression plasmid into HepG2 cells. Two days post transfection, the protein levels were determined by Western blot. The mean and standard deviations (n = 4) were presented. * and ** indicate P<0.05 and 0.01, respectively. (TIF) [file pone.0070001.s003.tif]

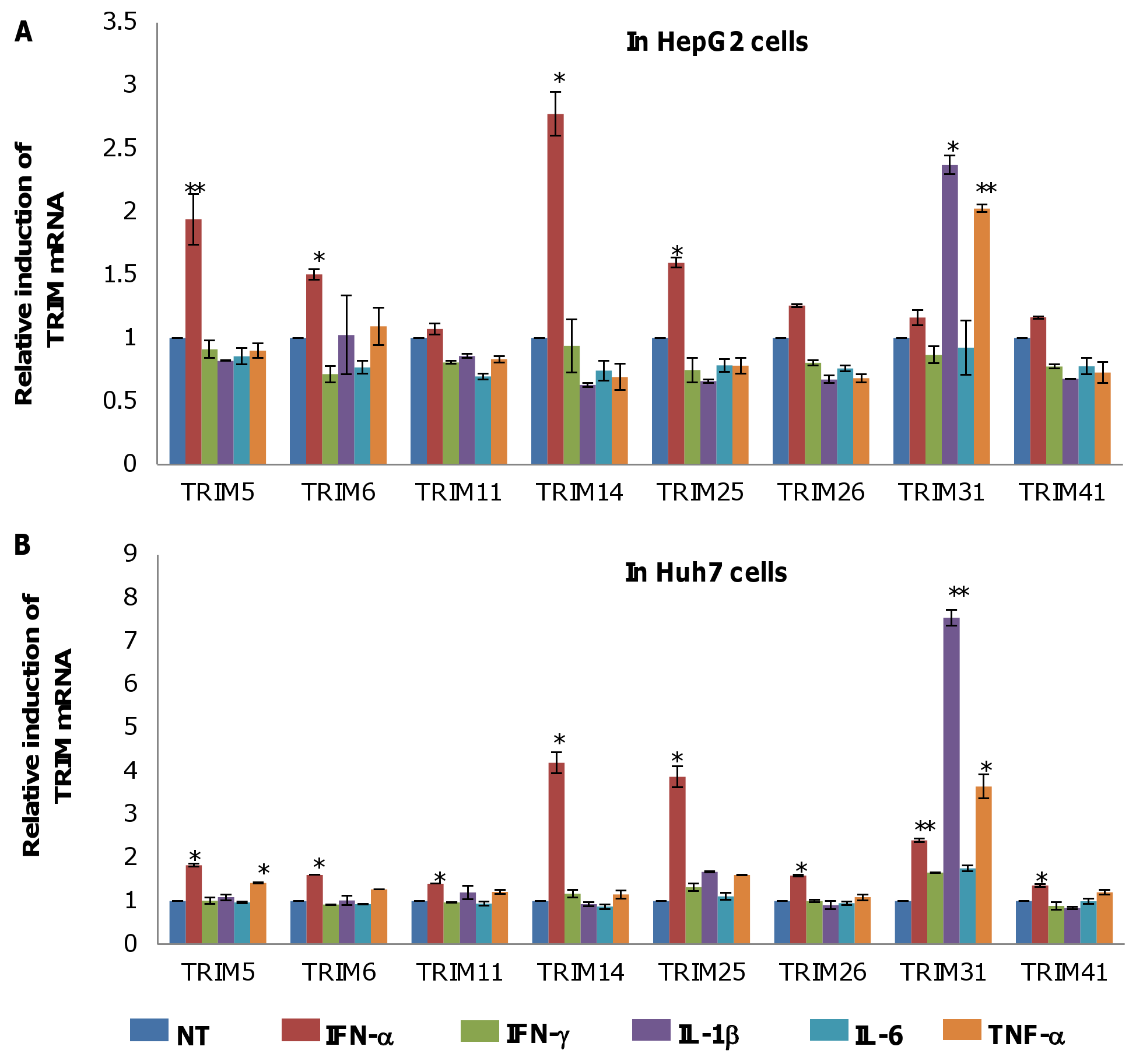

Supplement: Figure S4 — Effects of five representative inflammatory cytokines on the expression of the eight TRIMs that suppress HBV replication in human hepatoma cells. HepG2 (A) and Huh7 (B) cells were treated with 1000 IU/ml IFN-α, 10 ng/ml IFN-γ, 10 ng/ml IL-1β, 10 ng/ml IL-6, or 10 ng/ml TNF-α for 24 hours. The levels of the indicated TRIM mRNA were determined by a real-time RT-PCR assay. The mean and standard deviations (n = 3) were presented. * and ** indicate P<0.05 and 0.01, respectively. (TIF) [file pone.0070001.s004.tif]

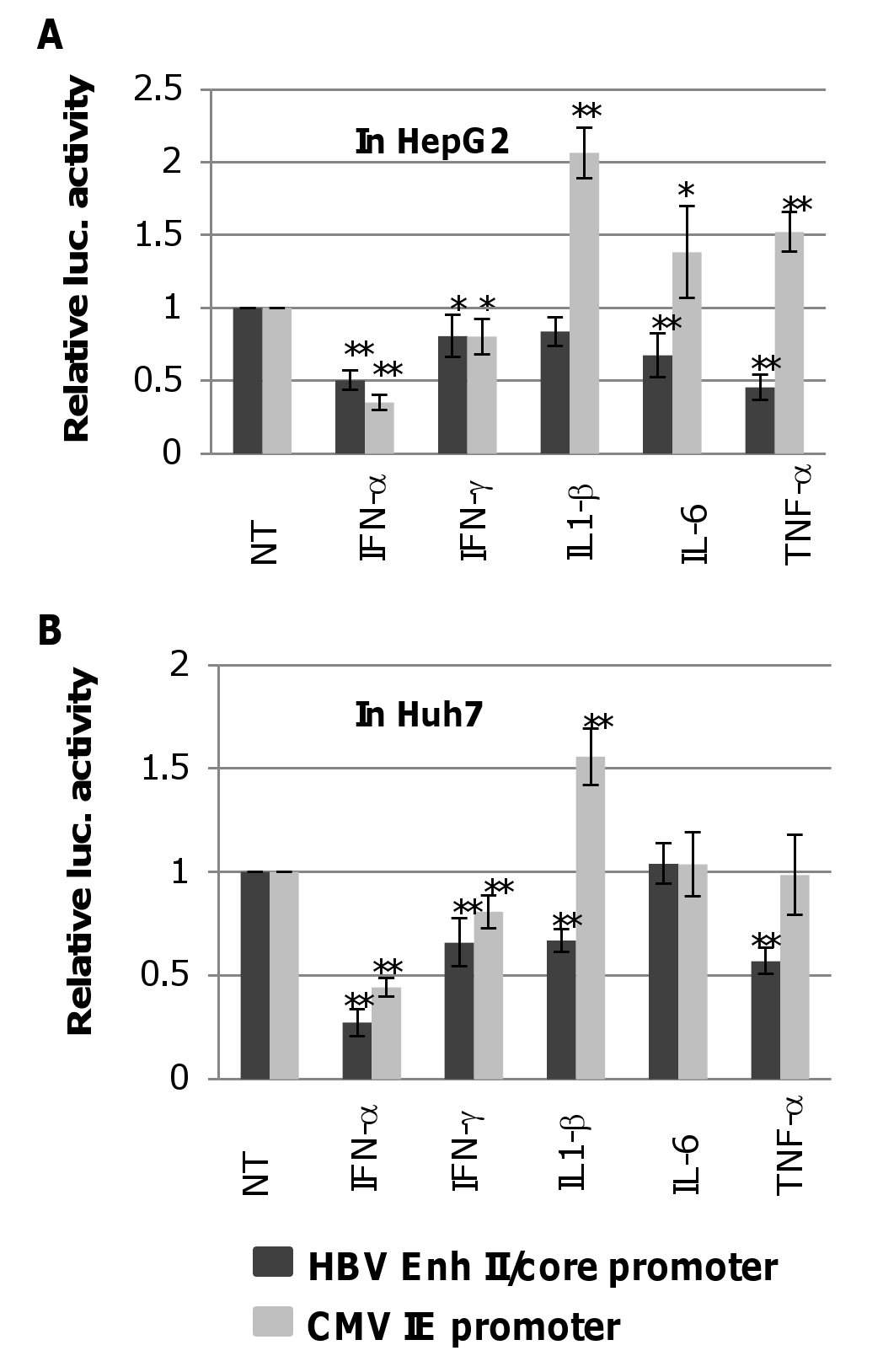

Supplement: Figure S5 — Effects of five representative inflammatory cytokines on HBV Enh II activity in human hepatoma cells. pGL4.10-HBV enhancer II was transfected into HepG2 cells with pCMV-renilla luciferase as an internal control. Six hours later, cells were treated with 1000 IU/ml IFN-α, 10 ng/ml IFN-γ, 10 ng/ml IL-1β, 10 ng/ml IL-6, or 10 ng/ml TNF-α for 48 hours. Luciferase activity was analyzed with a Dual-luciferase kit. The mean and standard deviations (n = 6) were presented. * and ** indicate P<0.05 and 0.01, respectively. (TIF) [file pone.0070001.s005.tif]
