# Supplementary material for: Identification and Characterization of Multiple TRIM Proteins That Inhibit Hepatitis B Virus Transcription
Source: PLoS One. 2013 Aug 1;8(8):e70001. doi: 10.1371/journal.pone.0070001 (PMC3731306; doi:10.1371/journal.pone.0070001)
Supplement: Table S1 — TRIM protein expression plasmids. (DOCX) [file pone.0070001.s006.docx]

**Supplemental Table S1.** TRIM protein expression plasmids.

| **TRIMs** | **Source** | **Accession number** | **Vector Type** |
| --- | --- | --- | --- |
| TRIM1 | Open Biosystems | MHS1010–58431 | pCMV-SPORT6 |
| TRIM2 | Open Biosystems | MHS1010–7429489 | pCMV-SPORT6 |
| TRIM3 | Origene | RC211928 | pCMV6-entry |
| TRIM4 | Origene | RC221925 | pCMV6-entry |
| TRIM5 | Origene | RC212800 | pCMV6-entry |
| TRIM6 | Origene | RC216413 | pCMV6-entry |
| TRIM8 | Origene | RC205812 | pCMV6-entry |
| TRIM11 | Origene | RC209630 | pCMV6-entry |
| TRIM14 | Origene | RC214697 | pCMV6-entry |
| TRIM15 | Open Biosystems | MHS1010–7508596 | pCMV-SPORT6 |
| TRIM16 | Origene | RC208564 | pCMV6-Entry |
| TRIM16L | Origene | RC214981 | pCMV6-Entry |
| TRIM18 | Open Biosystems | MHS1010–9205608 | pCMV-SPORT6 |
| TRIM21 | Open Biosystems | MHS1010–73808 | pCMV-SPORT6 |
| TRIM24 | GS | N/A | pcDNA3.1 (+) |
| TRIM25 | Open Biosystems | MHS1010–58392 | pCMV-SPORT6 |
| TRIM26 | Origene | RC202881 | pCMV6-entry |
| TRIM28 | Open Biosystems | MHS1010–9205415 | pCMV-SPORT6 |
| TRIM31 | Open Biosystems | MHS1010–74233 | pCMV-SPORT6 |
| TRIM32 | Origene | RC201289 | pCMV6-entry |
| TRIM34 | Origene | RC218306 | pCMV6-Entry |
| TRIM35 | Open Biosystems | MHS1010–73500 | pCMV-SPORT6 |
| TRIM37 | Origene | RC224302 | pCMV6-Entry |
| TRIM38 | Open Biosystems | MHS1010–7429568 | pCMV-SPORT6 |
| TRIM39 | Origene | RC213350 | pCMV6-entry |
| TRIM40 | Origene | RC223445 | pCMV6-entry |
| TRIM41 | Origene | RC210557 | pCMV6-entry |
| TRIM43 | Open Biosystems | MHS1010–74174 | pCMV-SPORT6 |
| TRIM44 | Open Biosystems | MHS1010–58247 | pCMV-SPORT6 |
| TRIM45 | Origene | RC215738 | pCMV6-entry |
| TRIM46 | Origene | RC223399 | pCMV6-entry |
| TRIM47 | Origene | RC218521 | pCMV6-entry |
| TRIM56 | Origene | RC200891 | pCMV6-Entry |
| TRIM58 | Origene | RC217706 | pCMV6-Entry |
| TRIM59 | Origene | RC221852 | pCMV6-Entry |
| TRIM62 | Origene | RC200126 | pCMV6-entry |
| TRIM69 | Origene | RC208173 | pCMV6-Entry |

GS: constructed by Genescript. N/A: not available.
