# Supplementary material for: Identification and Characterization of Multiple TRIM Proteins That Inhibit Hepatitis B Virus Transcription
Source: PLoS One. 2013 Aug 1;8(8):e70001. doi: 10.1371/journal.pone.0070001 (PMC3731306; doi:10.1371/journal.pone.0070001)
Supplement: Table S2 — Sequence of primers for real time PCR assays. (DOCX) [file pone.0070001.s007.docx]

**Supplemental Table S2.** Quantitative real-time PCR primers.

| **TRIMs** | **sequence** |  |
| --- | --- | --- |
| β-actin for | GCACAGAGCCTCGCCTT |  |
| β-actin rev | GTTGTCGACGACGAGCG |  |
| preC/C for | 5’-2275- TTCGCACTCCTCCAGCTTAT-2294-3’ |  |
| preC/C rev | 5’-2457-TCCCCACCTTATGAGTCCAA-2476-3’ |  |
| All HBV mRNA for | 5’-1545-CTCCCCGTCTGTGCCTTCTC-1564-3’ |  |
| All HBV mRNA rev | 5’-1883-GCCCCAAAGCCACCCAAG-1900-3’ |  |
| TRIM5 for | CTGGAGATGCTGAGGCAGAAGC |  |
| TRIM5 rev | GTCCAGGATGTCTCTCAGTTGC |  |
| TRIM6 for | CATTTGCTGGCTTTGTGAGCGG |  |
| TRIM6 rev | TTCCTGCTCCTCGTTCTTCAGC |  |
| TRIM11 for | CAGGATGCGTTGCTGTTCCAAG |  |
| TRIM11 rev | AAACGGCGAAGACGCTCGAACT |  |
| TRIM14 for | CAACAGGGTCTGGAGTATCAGC |  |
| TRIM14 rev | CTTGACGGGCTCAAAAGAGAGG |  |
| TRIM22 for | GGATCGTCAGTAGAGATGCTGC |  |
| TRIM22 rev | GAACTTGCAGCATCCCACTCAG |  |
| TRIM25 for | AAAGCCACCAGCTCACATCCGA |  |
| TRIM25 rev | GCGGTGTTGTAGTCCAGGATGA |  |
| TRIM26 for | GAACCACCTGAGTACCCTAAGG |  |
| TRIM26 rev | CTCAGCCACAATGTACTGCCTC |  |
| TRIM31 for | GAGCAGATCCAAGTCTTGCAGC |  |
| TRIM31 rev | CTCCTCTAGGACTTGATGCAGG |  |
| TRIM41 for | CCGAGAATCCAGGAGCCACAAA |  |
| TRIM41 rev | TCCAGGTGCTTCCTCAGTGGTT | |
